# REMOTE ELECTRONIC DATA CAPTURE OF MEDICATION USE AND AGITATION SYMPTOMS IN OLDER ADULTS WITH MCI OR DEMENTIA

**DOI:** 10.1093/geroni/igac059.1778

**Published:** 2022-12-20

**Authors:** Wan-Tai Au-Yeung, Lyndsey Miller, Sarah Gothard, Allison Lindauer, Jeffrey Kaye

**Affiliations:** Oregon Health and Science University, Portland, Oregon, United States; Oregon Health & Science University, Portland, Oregon, United States; Oregon Health & Science University, Portland, Oregon, United States; Oregon Health & Science University, Portland, Oregon, United States; Oregon Health & Science University, Portland, Oregon, United States

## Abstract

Timely and objective knowledge regarding behavioral and psychological symptoms (BPS) in older adults with cognitive impairment or dementia residing in the community setting is challenging to obtain. The Monitoring Dementia-Related Agitation using Technology Evaluation (MODERATE) Study aims to identify longitudinal changes of agitation and related symptoms for dyads living at home with dementia. To date, MODERATE has enrolled seven dyads (a person living with MCI or dementia and a spousal caregiver). The mean age of the participants is 75.7 (8.5) years, the age of the spousal caregivers is 71.0 (6.0) years, and 5 out of 7 of the participants with MCI or dementia are male. We created an online survey, which is sent weekly to the caregivers, to inquire of any changes in medication and frequency of symptoms of agitation experienced by the persons with MCI or dementia in the past week. As of 02/22/2022, 102 weekly surveys (mean per participant = 14.6) were sent out via email; 99 responses were returned (response rate of 97.1%). One caregiver used a personal computer (PC) only, 2 used smartphone only and 4 used both PC and smartphone to complete the surveys. The median time to complete the online survey was 3.83 minutes with IQR 1.72 – 9.14 minutes. “Complaining, negativism, refusal to follow directions” were the most commonly reported agitated behaviors reported (43% of surveys). Caregivers can provide regular detailed symptom profiles and medication reports online. This approach may be used for more timely and informative management of BPS in dementia.

